# The Children's Convalescent Home at Great Yarmouth

**Published:** 1888-08-18

**Authors:** 


### THE CHILDREN'S CONVALESCENT HOME AT GREAT YARMOUTH.

Miss Lucy Hansell sends us the following communication :—

It may not be generally known that there is a Children's Convalescent Home at Great Yarmouth ; so, perhaps a few particulars may be of use to benevolent persons, and also to hospitals. An annual subscription of £1 1s. will send a child to the Home for one month ; the only extra charge being 6*d*. a week for washing. Hospitals subscribing £21 annually can retain a "cot," and by this means can have a child all the year round at the Home. The Great Eastern Railway allow patients travelling to the Home to have return tickets for single fare. The return fare from London to Yarmouth is 5*s*. Children are received from two to twelve years of age. The Home stands near the beach, and faces the sea. It is replete with every comfort, and children benefit very greatly by the pure sea air and good food. The annual report can be had by application to the Hon. Secretary, J. B. T. Hales, Esq., Upper Close, Norwich. Subscriptions may be paid to him, and letters of recommendation would at once be sent to subscribers, and further particulars if desired.

